# Home-based brain–computer interface attention training program for attention deficit hyperactivity disorder: a feasibility trial

**DOI:** 10.1186/s13034-022-00539-x

**Published:** 2023-01-25

**Authors:** Choon Guan Lim, Chui Pin Soh, Shernice Shi Yun Lim, Daniel Shuen Sheng Fung, Cuntai Guan, Tih-Shih Lee

**Affiliations:** 1 Department of Developmental Psychiatry, Institute of Mental Health, 10, Buangkok View, Singapore, 539747 Singapore; 2 School of Computer Science and Engineering, Nanyang Technological University, Singapore, Singapore; 3 Neuroscience and Behavioral Disorders Program, Duke-National University of Singapore Graduate Medical School, Singapore, Singapore

**Keywords:** Attention deficit hyperactivity disorder, Attention training, EEG, Brain–computer interface, Child Behavior Checklist

## Abstract

**Background:**

Attention deficit hyperactivity disorder (ADHD) is a prevalent child neurodevelopmental disorder that is treated in clinics and in schools. Previous trials suggested that our brain–computer interface (BCI)-based attention training program could improve ADHD symptoms. We have since developed a tablet version of the training program which can be paired with wireless EEG headsets. In this trial, we investigated the feasibility of delivering this tablet-based BCI intervention at home.

**Methods:**

Twenty children diagnosed with ADHD, who did not receive any medication for the preceding month, were randomised to receive the 8-week tablet-based BCI intervention either in the clinic or at home. Those in the home intervention group received instructions before commencing the program and got reminders if they were lagging on the training sessions. The ADHD Rating Scale was completed by a blinded clinician at baseline and at week 8. Adverse events were monitored during any contact with the child throughout the trial and at week 8.

**Results:**

Children in both groups could complete the tablet-based intervention easily on their own with minimal support from the clinic therapist or their parents (at home). The intervention was safe with few reported adverse effects. Clinician-rated inattentive symptoms on the ADHD-Rating Scale reduced by 3.2 (SD 6.20) and 3.9 (SD 5.08) for the home-based and clinic-based groups respectively, suggesting that home-based intervention was comparable to clinic-based intervention.

**Conclusions:**

This trial demonstrated that the tablet version of our BCI-based attention training program can be safely delivered to children in the comfort of their own home.

*Trial registration* This trial is registered at clinicaltrials.gov as NCT01344044

**Supplementary Information:**

The online version contains supplementary material available at 10.1186/s13034-022-00539-x.

## Introduction

Attention deficit hyperactivity disorder (ADHD) is a prevalent neurodevelopmental disorder in children, and many individuals experience impairments such as disruptions to interpersonal relationships, psychosocial wellness and academic performance from preschool to adulthood [1–4]. Early identification and intervention have been shown to alter the trajectory of ADHD and prevent long-term negative consequences in individuals [5]. Intensive research over the past decades show that medication, psychosocial and behavioural interventions remain the leading evidence-based treatment approaches [3]. Yet, there is limited empirical evidence to support their long-term efficacy, in part due to the challenges of conducting such studies [6–8]. Promising alternative treatments, such as dietary interventions, cognitive training (including programmes such as Cogmed) and neurofeedback therapy are still in need of high-quality evidence to support their efficacy, while omega-3 fish oil supplementation has seen waning evidence of effect with better quality trials [9–14]. Very often, clinical treatment for ADHD does not involve one single intervention, but a multi-modal approach to address the multiple areas of needs.

Longitudinal studies have shown that inattentive symptoms of ADHD are more enduring and resistant to improvement over time compared to the hyperactive-impulsive symptoms [15, 16]. ADHD has been associated with aberrant brain wave activity that can be detected on the electroencephalogram (EEG) [17–19]. Neurofeedback therapy, which trains a person to modify their own EEG waves and thereby improve their ADHD symptoms, has been explored extensively among the children and adolescent population. Clinical trials reported sustained improvements in inattentive symptoms, while improvements in hyperactive/impulsive symptoms yielded mixed results [9, 20]. The therapy generally follows a protocol to modify specific EEG parameters with standard neurofeedback training protocols being theta/beta, sensori-motor rhythm and slow cortical potential [21].

We have harnessed the brain–computer interface (BCI) technology to develop a novel attention training program, using EEG as the interface between the individual and the computing device. Through a headband with two frontal dry EEG sensors, collected EEG signals are transmitted to the computer via Bluetooth-enabled protocol. The advanced signal processing techniques in the brain–computer interface analyses these EEG signals in relation to their state (e.g. resting) and task performance on the colour Stroop task [22], which then provides a model of EEG features that distinguishes between the individual’s attentive state and inattentive state. This enables a mathematical model to be developed that can compute their attention state at any point in time based on their EEG data. This attention level, which is fed back to the individual through a score (maximum of 100), is used to drive the game activities during training [23]. Our prior findings suggested that a training schedule comprising 24 sessions over 8 weeks could produce improvement in a child’s inattentive symptoms.

As clinic-based care is resource intensive for both the institution and the patient, we have articulated in our earlier reports that our eventual goal was to develop a home-based intervention to move the treatment of ADHD to the patients’ own home. There have been recent clinical trials reporting positive effects of game-based interventions in children with ADHD [24, 25]. We have since developed a tablet version of our BCI-training program with an industry partner, to whom our patented technology is licensed. In this trial, we aim to test the feasibility and safety of delivering this new tablet-based intervention at the child’s home without on-site support from any therapist.

## Methods

### Study design

**Fig. 1:**
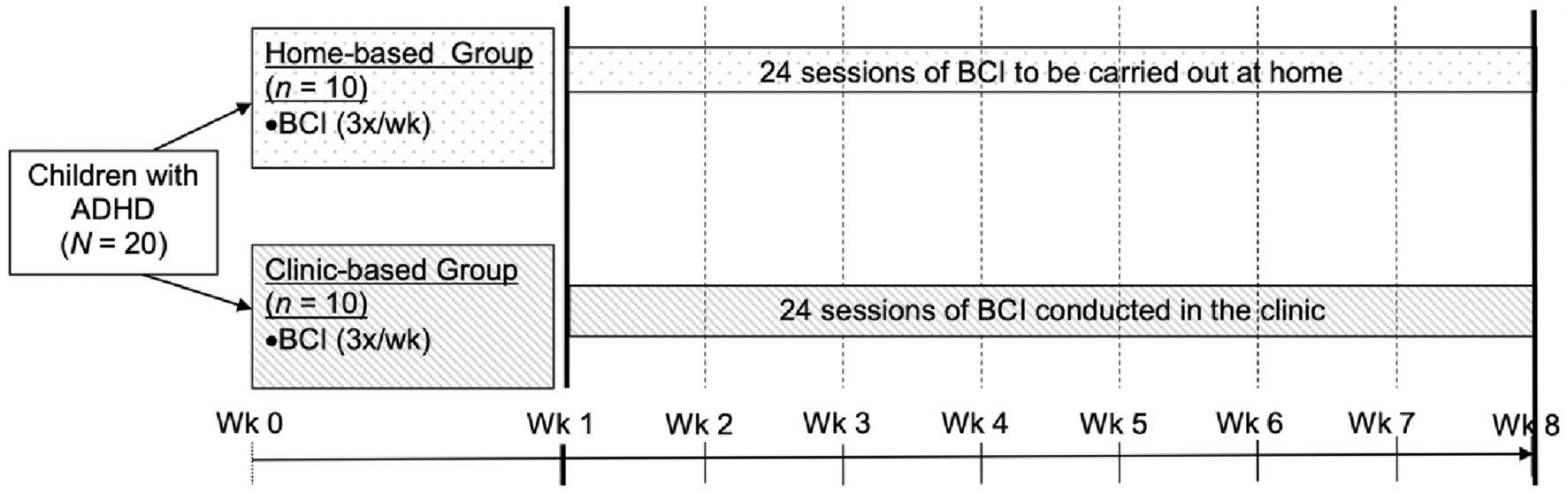
Gantt schedule illustrating study timeline and group allocation

We conducted a single-centre, outcome-assessor-blinded, parallel-group study at the Child Guidance Clinic, Institute of Mental Health in Singapore from 2019 to 2021. All participants underwent 24 BCI training sessions over a period of 8 weeks (Fig. 1).

### Participants

A total of 20 children aged 6 to 12 years old were recruited. All children were diagnosed with ADHD by a child psychiatrist based on the Diagnostic and Statistical Manual of Mental Disorders-Fourth Edition, text revised (DSM-IV TR) or the Diagnostic and Statistical Manual of Mental Disorders-Fifth Edition (DSM-5), and were referred by their attending doctor to participate in this study. Potential participants underwent a screening phase ascertain their eligibility for the trial. Parents completed the ADHD section of the Mini International Neuropsychiatric Interview for Children and Adolescents (MINI-KID) [26] and children fulfilled the criteria of either the predominantly inattentive or combined subtype of ADHD. None of the children screened fulfilled the criteria of predominantly hyperactive subtype of ADHD. The Kaufman Brief Intelligence Test, Second Edition (KBIT-2) [27] was administered to children whose parent(s) reported that they were failing English and/or Mathematics in their recent school test. This was conducted to ascertain whether they have the language ability to understand the questionnaires and game instructions. No children were excluded based on their KBIT-2 scores. Children on any medication (i.e., stimulants, atomoxetine and traditional Chinese medicine) or supplements (e.g. omega-3 oil, flax seed oil, cod liver oil) underwent a washout period of 1 and 3 months respectively. Children did not undergo any psychosocial treatment or behavioural intervention while on the trial, and none of them had a history of receiving brain–computer interface or neurofeedback intervention. We excluded children with co-morbid severe psychiatric condition, known sensory-neural deficit, epileptic seizures and intellectual disability (i.e. IQ 70 and below).

### Procedure

**Fig. 2:**
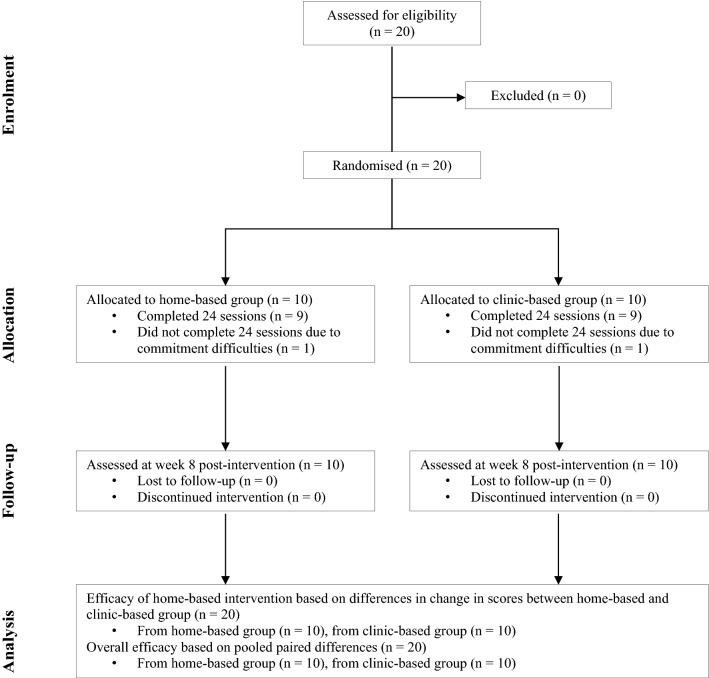
Participant flowchart (CONSORT flow diagram)

Participants were randomly assigned to either the clinic-based group or the home-based group (Fig. 2) using the opaque sealed envelope method. A random allocation sequence was generated using the permuted block technique to assign the participants to either group at a 1:1 ratio. Participants assigned to the clinic-based group received the intervention under the supervision of a study administrator, while participants in the home-based group received the intervention under the supervision of their parents.

For the home-based group, a study administrator briefed and guided parents through the set-up procedure during the baseline visit (week 1) to ensure proper administration of the intervention at home. Study team members then followed-up via phone calls on week 3, 5 and 7 to check on the progress of the home-based group. In addition, the game software was programmed to unlock three sessions weekly to ensure that the home-based group is receiving the same amount of training per week as those in the clinic-based group.

Prior to and after the intervention programme, both parents and child attended a teleconsultation with the clinician via Zoom Meetings, where the clinician will assess for changes in ADHD symptoms and adverse events.

### Intervention programme

The BCI system consisted of a headband with dry EEG electrode sensors and a tablet. Similar to earlier trials [28, 29], we adopted an 8-week training programme, consisting of thrice-weekly sessions (24 sessions in total).

#### Calibration

Prior to playing the game at baseline (week 1) and after completion of the training programme at post-intervention (week 8), participants underwent calibration, where they were required to complete the colour Stroop task on the tablet to generate an individualised attention model, which was used to predict their attention level online during the game play.

#### Cogoland

**Fig. 3:**
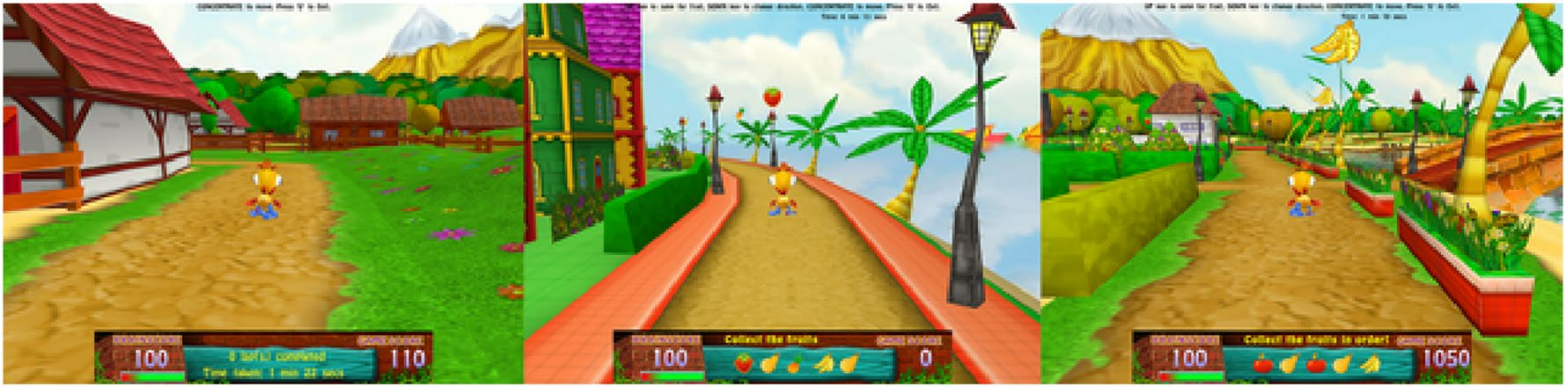
Cogoland game interface [28]

Cogoland is a 3D computerized graphic game developed locally with the intention to train attention [28]. This BCI-based intervention is available as an application software that can be downloaded onto a tablet. During the training sessions, brainwaves detected by the headband are transmitted to the tablet, analysed by the game system’s algorithm, and translated into quantifiable attention scores. Their attention scores, ranging from 0 (minimum attention) to 100 (maximum attention), were reflected on the tablet screen, providing real-time feedback to participants about their attention level. In other words, the higher the participant’s attention score, the faster the avatar’s movement. The game consisted of 3 levels. In level 1, the main goal was to cover as much distance as possible within 10 min and achieve a high score. Level 2 required participants to collect fruits by pressing the jump button at the correct timing, and level 3 required collecting the fruits in a specific order (Fig. 3).

At every alternate session, participants were required to answer 20 English and Mathematics questions after completing the training segment. This aims to help participants generalise the learnt ability to regulate their attention from the game to daily academic tasks.

### Assessments

The following questionnaires were administered at baseline (week 1) and post-intervention (week 8) to assess for treatment outcomes. The ADHD Rating Scale (ADHD-RS) [30] is an 18-item rating scale that measures ADHD symptoms in children and adolescents based on the DSM-IV TR. Both parents and the clinician rated the frequency of both inattention and hyperactive-impulsive symptoms on a 4-point Likert scale (1 = never or rarely, 4 = very often). The Child Behaviour Checklist (CBCL) [31] is a 113-item parent-rated questionnaire which yields information on behavioural and emotional problems in children and adolescents. Scores are summarized into the following domains: aggressive behaviour, anxious/depressed mood, attention problems, rule breaking behaviour, social problems, somatic complaints, thought problems, withdrawn/depressed mood, internalizing problems, externalizing problems and total problems, as well as the DSM-oriented scores which includes affective, anxiety, attention deficit/hyperactivity, oppositional, conduct and somatic problems. Parents are required to rate their child’s behavior on a 3-point Likert scale (0 = not true, 2 = very true or often true). For the purpose of this study, scores from the following domains were analysed: attention problems, internalizing problems, externalizing problems, total problems, and attention deficit/hyperactivity behaviours.

During teleconsultations, the clinician will rate the child based on the Children’s Global Assessment Scale (CGAS) and the Clinical Global Impressions Scale (CGI). The CGAS provides a single global rating on a scale of 1 to 100, reflecting the child’s overall functioning which ranges from “needs constant supervision” (scores 1–10) to “superior functioning” (scores 91–100). The CGI comprises of 2 companion 7-point measures evaluating the severity of psychopathology (1 = normal/not mentally ill at all, 7 = extremely mentally ill) and global improvement (1 = very much improved, 7 = very much worse).

### Outcomes

Primary outcome measures consisted of treatment completion and adherence rates, adverse events and qualitative feedback obtained from participants and parents. The secondary outcome was the short-term effect in parent-rated and clinician-rated ADHD-RS scores, and CBCL attention, internalizing, externalizing, total and ADHD problems t-scores.

### Blinding

While participants, their parents, and study administrators were aware of the treatment allocation, the clinician was kept blinded throughout the trial. Participants and their parents were regularly advised not to inform the clinician their group allocation.

### Data analysis

To address the primary hypothesis of evaluating the feasibility of conducting BCI training sessions at home, treatment completion rates were calculated as the percentage of participants who had completed a minimum of 20 sessions in 8 weeks as per protocol. Qualitative feedback was collected from participants and their parents to assess for technical issues and other difficulties encountered during the trial. Participants and their parents met the clinician before and after completing the BCI training, and any adverse events was recorded by the clinician.

In order to explore the efficacy of home-based BCI training, the Mann–Whitney U test was used to analyse the difference in change in ADHD-RS and CBCL t-scores between the home-based group and clinic-based group. Analyses were performed on an intent-to-treat basis; all participants were analysed according to the group they were randomised to regardless of their compliance with protocol. As the sample size is small, non-parametric tests were used. All tests were two-tailed and performed at a 5% level of significance. Statistical analyses were implemented using the Statistical Package for the Social Sciences version 26 (SPSS 26).

## Results

### Study participation

A total of 20 participants (M_age_ = 9.93, SD = 1.69) were recruited in the study, including 16 males (80%) and 4 females (20%). Table 1 provides a summary of their baseline demographic and clinical characteristics. Both groups were found to be comparable in all their baseline characteristics.

**Table 1:** Baseline socio-demographic and clinical characteristics

	Total (n = 20)	Home (n = 10)	Clinic (n = 10)	*p*
Age				
Mean (SD)	9.93 (1.69)	10.33 (1.97)	9.54 (1.33)	0.35
Gender				
Male/female	16/4	6/4	10/0	0.14
ADHD subtype				
Inattentive/combined	11/9	4/6	7/3	0.28
Comorbid neurodevelopmental conditions	2 (10%)	1 (10%)	1 (10%)	
Dyslexia	1	0	1	–
Tourette syndrome	1	1	0	
Children Global Assessment Scale (CGAS)				
n	19	9	10	
Mean (SD)	58.53 (4.48)	58.22 (3.77)	58.8 (5.22)	0.68
Clinical Global Impression-Severity (CGI-S)				
Mean (SD)	4.2 (0.49)	4.3 (0.67)	4.0 (0.00)	0.28
ADHD-RS inattention score				
Clinician-rated, Mean (SD)	17.1 (5.56)	17.9 (5.61)	16.1 (5.69)	0.85
Parent-rated, Mean (SD)	16.8 (5.72)	17.7 (4.32)	15.9 (6.97)	0.39
ADHD-RS hyperactivity score				
Clinician-rated, Mean (SD)	11.7 (6.83)	12.5 (5.72)	10.9 (8.16)	0.74
Parent-rated, Mean (SD)	12.0 (6.89)	12.2 (5.37)	11.8 (8.44)	0.68
Child Behavior Checklist (CBCL)				
Attention problems, Mean (SD)	71.6 (11.73)	75.8 (12.19)	67.3 (10.09)	0.14
Internalizing problems, Mean (SD)	58.2 (9.98)	62.0 (7.56)	54.3 (10.95)	0.08
Externalizing problems, Mean (SD)	58.9 (10.91)	60.1 (10.10)	57.6 (12.08)	0.53
Total problems, Mean (SD)	63.4 (8.32)	66.1 (6.44)	60.7 (9.42)	0.14
Attention deficit hyperactivity problems, Mean (SD)	66.2 (8.47)	67.4 (8.98)	64.9 (8.21)	0.53

### Primary outcome

The primary outcome measures of feasibility are displayed in Table 2, with a summary of the outcomes described below.

**Table 2:** Feasibility outcome measures comprising of completion and retention rates, session spread, difficulties faced and adverse events

	Total	Home	Clinic
Completion and retention			
Treatment completion (at least 20 sessions)	95% (19/20)	95% (9/10)	95% (9/10)
Treatment completion (all 24 sessions)	90% (18/20)	95% (9/10)	95% (9/10)
Retention (post-intervention)	100% (20/20)	100% (10/10)	100% (10/10)
Session spread			
3 sessions/week throughout trial	10% (2/20)	10% (1/10)	10% (1/10)
More than 3 weeks of having 3 sessions/week	75% (15/20)	60% (6/10)	90% (9/10)
Technical issues			
Headband disconnectivity	30% (6/20)	30% (3/10)	30% (3/10)
Game application software hanging/crashing	0% (0/20)	0% (0/10)	0% (0/10)
Gameplay defect	0% (0/20)	0% (0/10)	0% (0/10)
Other difficulties			
Game set up by parents^a,b^	–	20% (2/10)	–
Game set up by child^a,b^	–	0% (0/10)	–
Require parent supervision^a,b^	–	20% (2/10)	–
Gameplay^b^	25% (5/20)	20% (2/10)	30% (3/10)
Adverse events			
Serious adverse events	0	0	0
Sleep problems	0	0	0
Somatic complaints	5% (1/20)	10% (1/10)	0
Other complaints^c^	5% (1/20)	0	10% (1/10)

^a^Feedback of parents/child for home-based group

^b^Provided qualitative feedback of difficulty

^c^Pre-existing vocal tics became more frequent on days the child played the game

#### Completion and retention rates

All participants except one completed the minimum of 20 BCI sessions within 8 weeks. Two participants (10%), one from each treatment group, were unable to complete all 24 sessions due to scheduling difficulties. The participant from the home-based group missed 13 sessions (54%) despite periodic reminder calls to the parent. The post-intervention feedback from the parent indicated that it was difficult to continue with the training once schoolwork piled up and that the child lost momentum in playing the game after getting frustrated from the headband disconnecting often. As for the participant from the clinic-based group, he/she missed 4 sessions (17%) because the parent had difficulties finding someone to accompany the child to the clinic due to work commitments. All participants attended the assessment visits at baseline, pre- and post-intervention.

#### Session spread

A uniform spread of three sessions per week for the BCI training was adhered by two (10%) of the participants, one from each group. Fifteen participants (75%) had more than 3 weeks (out of 9) of having a uniform spread of three sessions per week, of which six and nine children were from the home-based and clinic-based group respectively. The session spreads for each participant are displayed in Additional file 1: Table S1.

#### Technical issues and other difficulties

Three participants (30%) in each of the treatment groups experienced difficulties with the headband connectivity, which affected their enjoyment of playing the game as the screen would freeze whenever the headband signal was lost. No other technical issues were reported.

With respect to the home-based group, two parents (20%) experienced some difficulties with setting up the game tablet and headset, however none of the children reported the same issue. While 80% of the parents found that their child could undergo the BCI training with little to no supervision, two of them indicated having to supervise their child during training. One was because their child would forget to play the game consistently, while the other was because the child would get frustrated with the headband disconnecting frequently.

In terms of overall gameplay, five participants (25%) found the intermediate and hard levels to be somewhat challenging however that did not deter them from completing the training. Of the five, two were from the home-based group while the rest were from the clinic-based group.

#### Adverse events

Two (10%) participants reported experiencing an adverse event after completing the sessions. One indicated feeling mildly fatigued after playing the game, while another reported that the child’s pre-existing vocal tics were more frequent on days that the game was played. None of these two adverse events required medical treatment or was rated to be severe. In both cases, the participants were able to carry on with the intervention sessions.

### Changes in clinician- and parent-rated ADHD-RS

The description and analysis of clinician- and parent-rated ADHD-RS scores are presented in Table 3. For the clinician-rated ADHD-RS, mean change (improvement) of inattentive symptom scores were not significantly different between home-based (M_change_ = 3.2) and clinic-based (M_change_ = 3.9) group, *U* = 42.0, *z* = 0.179, *p* = 0.90, *r* = 0.04. Similarly, parent-rated ADHD-RS inattentive symptoms scores were also not significant between home-based (M_change_ = 3.0) and clinic-based (M_change_ = 1.8) group, *U* = 39.5, *z* = − 0.798.179, *p* = 0.44, *r* = − 0.19.

**Table 3:** Comparison of change in ADHD-RS scores between week 0 and week 8 for home-based and clinic-based groups

	Home (Mean, SD)	Clinic (Mean, SD)	*U*	*z*	*p*	*r*
Clinician-rated inattention score						
Week 0	17.9 (5.61)	16.1 (5.69)				
Week 8	14.7 (4.97)	14.0 (6.30)	42.0	0.179	0.90	0.04
Change	− 3.2^a^ (6.20)	− 3.9^a^ (5.08)				
Parent-rated inattention score						
Week 0	17.7 (4.32)	15.9 (6.97)				
Week 8	14.7 (4.97)	14.1 (6.31)	39.5	− 0.798	0.44	− 0.19
Change	− 3.0^a^ (4.24)	− 1.8^a^ (4.39)				
Clinician-rated hyperactivity score						
Week 0	12.5 (5.72)	10.9 (8.16)				
Week 8	11.2 (6.23)	10.3 (5.02)	46.0	0.541	0.63	0.12
Change	− 1.3^a^ (4.17)	− 2.5^a^ (4.34)				
Parent-rated hyperactivity score						
Week 0	12.2 (5.37)	11.8 (8.44)				
Week 8	11.4 (6.26)	9.7 (5.29)	60.5	0.804	0.44	0.19
Change	− 0.8^a^ (3.74)	− 2.1^a^ (4.15)				

^a^Negative mean change scores indicate improvement in reported symptoms

The same pattern of results was found for ADHD-RS hyperactivity subscale for clinician-rated, *U* = 46.0, *z* = 0.541, *p* = 0.63, *r* = 0.12, and parent-rated scores, *U* = 60.5, *z* = 0.804, *p* = 0.44, *r* = 0.19. These findings indicate that the home-based group was comparable to the clinic-based group in terms of improvement of symptom scores after completing the BCI training.

#### Changes in other secondary outcome measures

The mean change (improvement) for the following scales have been summarized in Table 4; clinician-rated CGAS and CGI-S as well as parent-rated ADHD-RS and CBCL subscales scores. The outcomes for all the measures were consistent in their findings that there were no significant differences between home-based and clinic-based groups in terms of improvement in scores, *p* = 0.68 to 1.00, *r* = − 0.05 to 0.09.

**Table 4:** Change in secondary outcome measures of CGAS, CGI-S and CBCL subscale scores between week 0 and week 8

	Home	Clinic	*U*	*z*	*p*	*r*
Clinician-rated CGAS						
n	10	10				
M_change_ (SD)	5.56 (3.68)	3.70 (7.88)	50.0	0.412	0.72	0.09
Clinician-rated CGI-S						
M_change_ (SD)	− 0.06^a^ (0.70)	− 0.50^a^ (0.53)	47.5	− 0.213	0.85	− 0.05
Parent-rated CBCL attention problems						
M_change_ (SD)	− 5.00^a^ (7.82)	− 3.70^a^ (8.11)	45.5	− 0.341	0.74	− 0.08
Parent-rated CBCL internalizing problems						
M_change_ (SD)	− 1.50^a^ (7.09)	− 3.10^a^ (7.88)	55.5	0.418	0.68	0.09
Parent-rated CBCL externalizing problems						
M_change_ (SD)	− 2.70^a^ (5.79)	− 3.60^a^ (6.22)	53.5	0.265	0.80	0.06
Parent-rated CBCL total problems						
M_change_ (SD)	− 3.00^a^ (5.06)	− 3.80^a^ (4.19)	51.0	0.076	1.00	0.02
Parent-rated CBCL ADHD problems						
M_change_ (SD)	− 1.90^a^ (6.21)	− 2.30^a^ (5.46)	50.0	0.000	1.00	0.00

^a^Negative mean change scores indicate improvement in reported symptoms

## Discussion

Home-based psychological treatments are increasingly in demand to meet the treatment gap in resource-limited contexts such as high disease burden [32], lack of trained mental health professionals in low- and middle-income countries [33], and low allocation of resources and logistic limitations in clinical settings [34, 35]. In this study we examined the feasibility of a home-based BCI intervention for children with ADHD. Our results were encouraging and suggested that the BCI-based attention training program could be delivered via tablet at the patient’s own home without on-site therapist support. Although there is a dearth of research in home-based BCI interventions for ADHD, our findings are consistent with results from other independent home-based EEG neurofeedback interventions targeting chronic pain in adults [36], recovery of motor control after stroke [37] and social competence for college students with autism spectrum disorder (ASD) [38]. However, it should be noted that these studies are not directly comparable to our own given the difference in study population.

Nearly all the children completed a minimum of 20 training sessions within 8 weeks. Qualitative feedback received from some parents suggested that several children even managed their own training without needing support from their parents. It was also reported that a few of them did not require reminder from their parents to log in for the training sessions. These findings suggest that the intervention was engaging enough to motivate young children to follow through with the training schedule independently. Adverse events, in line with our earlier findings, were uncommon and mild [23, 29]. Unlike our earlier trials however, no children complained of headache. The child who experienced worsening of tics was anomalous and possibly related to the child’s emotions during the training session.

While the main goal of this study was not to test clinical efficacy, some relevant effects were observed. Participants in both home-based and clinic-based groups showed comparable improvements in the clinician- and parent-rated inattentive symptom scores on the ADHD-RS. Though the improvements in this study were not clinically significant, short-term efficacy of the clinic-based intervention was found in our previous randomized controlled trial (RCT) with 172 children diagnosed with inattentive or combined subtypes of ADHD [28].

This trial indicated several areas of improvement that will be needed for the equipment and hardware. Improved sturdiness of the headband and its connectivity will help sustain enjoyment during gameplay. Children may become frustrated and lose their interest if there are frequent interruptions to the game. Implementing a strategy to remind parents and the children about upcoming sessions, accompanied with in-game rewards for completing the scheduled sessions may improve compliance with the training protocols.

Being a neurodevelopmental condition, ADHD symptoms typically presents during formative years, and this creates a need for appropriate early interventions [5, 39]. Direct intervention with these young children is challenging due to their developing language and cognitive abilities. Games provide a viable way to engage them in learning executive functioning skills and can be an adjuvant treatment to medication [40–43]. Early intervention before the accumulation of more severe impairment also improves the chance of a better outcome and can hopefully alter the course of an otherwise chronic condition [5].

It is important to note the limitations of this small pilot trial. First, participants who volunteered to join our study were likely to be more motivated. They were also more likely to have milder ADHD symptoms as they were required to be off medications and supplements throughout the entire duration of the trial. Second, study participants were not blinded and even though the clinician was blinded to the group allocation, the clinician was aware that all participants received treatment. Third, our primary aim was to assess the feasibility of this home-based approach. Even though we collected and presented some clinical outcomes (such as CBCL rating scale scores), this study was not adequately powered to evaluate the clinical efficacy of the intervention. We did however decide to exclude co-interventions to assess if the degree of improvement was comparable to our previous trials. This allows us to also remove the influence co-interventions may have on the adverse effects or treatment compliance. Fourth, we did not collect participant information on past psychosocial and/or behavioural intervention thus we were not able to control for this. However, it is likely that this would have minimal effect on our ability to examine the feasibility of carrying out the home-based intervention without on-site support from any therapist. Lastly, our study administrators also provided reminder calls to parents, which would not be available outside of this study. Given that they likely helped with treatment compliance especially for the home-based group, incorporating consistent follow up calls might be necessary for home-based interventions to run effectively.

## Future research

As a relatively new form of therapy, more research still needs to be done on BCI-based interventions to cement its position as a reliable alternative treatment for ADHD. Future research can compare the effectiveness of BCI-based interventions against traditional treatment options such as pharmacologic and behavioural therapy. In addition, clinical trials can be conducted with a waitlist-control group to further examine its therapeutic efficacy in both clinic and home settings. A placebo-control group can also be included to account for the placebo effect. Lastly, future research could explore the use of BCI-interventions for other psychiatric conditions like anxiety disorder and neurodevelopmental conditions like autism.

## Conclusion

Our earlier RCT has shown that our BCI-based attention training programme can improve inattentive symptoms in ADHD. This study’s findings suggest that the tablet-based version of our brain–computer interface attention training program can be delivered in the patients’ own home safely without on-site therapist supervision and with minimal technical support, offering an additional treatment option to patients without further stretching the limited resources of clinic-based care. Furthermore, this home-based training can be a viable option for parents seeking to alleviate inattentive symptoms in their children with ADHD as early as during preschool.

## Supplementary Information

**Additional file 1: Table S1.** Session spreads for each participant in both the clinic- and home-based groups.

## Data Availability

The datasets analyzed during the current study are available from the corresponding author on reasonable request.
