# Supplementary material for: Home-based brain–computer interface attention training program for attention deficit hyperactivity disorder: a feasibility trial
Source: Child Adolesc Psychiatry Ment Health. 2023 Jan 25;17:15. doi: 10.1186/s13034-022-00539-x (PMC9878772; doi:10.1186/s13034-022-00539-x)
Supplement: Supplementary file 1 — Additional file 1: Table S1. Session spreads for each participant in both the clinic- and home-based groups. [file 13034_2022_539_MOESM1_ESM.pdf]

Additional file 1

Table S1. Session spreads for each participant in both the clinic- and home-based groups

Group	Participant	Number of sessions completed per week									Total
		Week 1	Week 2	Week 3	Week 4	Week 5	Week 6	Week 7	Week 8	Week 9	
Clinic-based	C1	2	3	2	3	3	4	3	3	1	24
	C2	3	3	3	3	1	3	3	3	2	24
	C3	1	3	1	2	1	3	3	3	3	20
	C4	1	3	3	3	3	2	3	3	3	24
	C5	3	3	3	3	3	3	3	2	1	24
	C6	3	3	3	3	3	3	3	3		24
	C7	2	3	3	2	3	3	3	3	2	24
	C8[a]	1	3	5	5	2	3	2	2	1	24
	C9	3	1	3	3	3	3	3	2	3	24
	C10	1	2	3	3	3	3	2	3	4	24
Home-based	H1	2	1	3	2	3	3	2	5	3	24
	H2[a]	3	5	5	2	3	3	2	1		24
	H3	3	3	3	2	4	3	1	4	1	24
	H4	2	3	3	1	3	3	4	3	2	24
	H5	3	3	3	3	3	3	3	3		24
	H6	2	4	5	3	3	3	3	1		24
	H7[a]	3	3	2	1	4	2	3	4	2	24
	H8[a]	3	2	2	4	3	2	5	3		24
	H9	3	3	3	2	3	3	1	3	3	24
	H10[a]	2	2	0	0	0	3	0	2	2	11

[a]Participants who did not have more than 3 weeks of completing a uniform spread of three sessions per week.
